# P101 If the inputs are flawed, so will be the outputs: a rule-based system towards reliable microbiology data

**DOI:** 10.1093/jacamr/dlaf230.108

**Published:** 2025-12-04

**Authors:** Sana Boujaafar, Kezia Taylor, Fred Mutisya, Philippe Berger, Taïoh Yokoyama, Mathieu Raad, Catrin E Moore

**Affiliations:** SmartbioticⓇ, France; Faculty of Pharmacy, University of Monastir, Tunisia; Infection and Immunity, St George’s School of Health and Medical Sciences, City St George’s, University of London, London, UK; SmartbioticⓇ, France; Moi University, Nairobi, Kenya; SmartbioticⓇ, France; SmartbioticⓇ, France; SmartbioticⓇ, France; Sarrebourg Hospital, Sarrebourg, France; Infection and Immunity, St George’s School of Health and Medical Sciences, City St George’s, University of London, London, UK

## Abstract

**Background:**

High-quality microbiology data are critical to inform antimicrobial stewardship, resistance surveillance and clinical decision-making. Yet, the utility of many existing microbiology datasets is impeded by considerable variability in clinical sampling and laboratory practices along with demonstrable inconsistencies in antimicrobial susceptibility testing (AST) data and reporting. This study, conducted within the Comprehensive Understanding of Disease and AI Research (CURE) project in collaboration with City St George’s and the University of Leeds, aims to verify and validate a rule-based system developed by SmartBiotic^®^ to evaluate microbiological data prior to analysis.

**Methods:**

The data quality assessment was conducted according to the guidelines of EUCAST and CLSI, which describe the criteria for resistance and susceptibility of bacterial phenotypes. We developed a rule-based system incorporating these standardized criteria which we validated using existing laboratory data. Three performance indicators were established: (i) the number of conflicts between our rules based application and real-world data, identifying potential laboratory errors, (ii) the percentage of laboratory tests performed for which the rule had already provided the answer, and (iii) the percentage of inferred results that could be generated by the system to evaluate dataset enrichment capabilities.

**Results:**

A total of 539 bacteriological rules were identified from EUCAST and CLSI. From 1138 antibiograms, 17 051 antibiotic susceptibility tests were produced in the laboratory which were analysed in our system. Of these, 84 results (5‰) were found to be in conflict with the rules, while 231 redundancies with the rules were detected. In addition, 11 264 additional results could be inferred using the available data together with the rules, this led to a 66% expansion of the database.

**Conclusions:**

The rule-based algorithm according to EUCAST and CLSI guidelines successfully integrates the concerted effort to enhance the reporting and analysis of available human clinical microbiology data; this set of rules can be used to harmonize global data standards. The system demonstrates a practical utility for identifying data inconsistencies and enriching datasets through validated inference mechanisms. Expert validation by clinical microbiologists and infectious disease specialist consultations are planned to further refine system applicability.

